# White matter damage due to vascular, tau, and TDP-43 pathologies and its relevance to cognition

**DOI:** 10.1186/s40478-022-01319-6

**Published:** 2022-02-05

**Authors:** Sheelakumari Raghavan, Scott A. Przybelski, Robert I. Reid, Timothy G. Lesnick, Vijay K. Ramanan, Hugo Botha, Billie J. Matchett, Melissa E. Murray, R. Ross Reichard, David S. Knopman, Jonathan Graff-Radford, David T. Jones, Val J. Lowe, Michelle M. Mielke, Mary M. Machulda, Ronald C. Petersen, Kejal Kantarci, Jennifer L. Whitwell, Keith A. Josephs, Clifford R. Jack, Prashanthi Vemuri

**Affiliations:** 1 Department of Radiology, Mayo Clinic, 200 First Street SW, Rochester, MN 55905 USA; 2 Department of Quantitative Health Sciences, Mayo Clinic, Rochester, MN 55905 USA; 3 Department of Information Technology, Mayo Clinic, Rochester, MN 55905 USA; 4 Department of Neurology, Mayo Clinic, Rochester, MN 55905 USA; 5 Department of Neuroscience, Mayo Clinic, Jacksonville, FL 32224 USA; 6 Department of Laboratory Medicine and Pathology, Mayo Clinic, Rochester, MN 55905 USA; 7 Department of Psychiatry and Psychology, Mayo Clinic, Rochester, MN 55905 USA

**Keywords:** Diffusion tensor imaging, Neurite dispersion density imaging, Cerebrovascular disease, Tau positron emission tomography, TAR DNA binding protein of 43 kDa

## Abstract

Multi-compartment modelling of white matter microstructure using Neurite Orientation Dispersion and Density Imaging (NODDI) can provide information on white matter health through neurite density index and free water measures. We hypothesized that cerebrovascular disease, Alzheimer’s disease, and TDP-43 proteinopathy would be associated with distinct NODDI readouts of white matter damage which would be informative for identifying the substrate for cognitive impairment. We identified two independent cohorts with multi-shell diffusion MRI, amyloid and tau PET, and cognitive assessments: specifically, a population-based cohort of 347 elderly randomly sampled from the Olmsted county, Minnesota, population and a clinical research-based cohort of 61 amyloid positive Alzheimer’s dementia participants. We observed an increase in free water and decrease in neurite density using NODDI measures in the genu of the corpus callosum associated with vascular risk factors, which we refer to as the vascular white matter component. Tau PET signal reflective of 3R/4R tau deposition was associated with worsening neurite density index in the temporal white matter where we measured parahippocampal cingulum and inferior temporal white matter bundles. Worsening temporal white matter neurite density was associated with (antemortem confirmed) FDG TDP-43 signature. Post-mortem neuropathologic data on a small subset of this sample lend support to our findings. In the community-dwelling cohort where vascular disease was more prevalent, the NODDI vascular white matter component explained variability in global cognition (partial R^2^ of free water and neurite density = 8.3%) and MMSE performance (8.2%) which was comparable to amyloid PET (7.4% for global cognition and 6.6% for memory). In the AD dementia cohort, tau deposition was the greatest contributor to cognitive performance (9.6%), but there was also a non-trivial contribution of the temporal white matter component (8.5%) to cognitive performance. The differences observed between the two cohorts were reflective of their distinct clinical composition. White matter microstructural damage assessed using advanced diffusion models may add significant value for distinguishing the underlying substrate (whether cerebrovascular disease versus neurodegenerative disease caused by tau deposition or TDP-43 pathology) for cognitive impairment in older adults.

**Graphical abstract:**

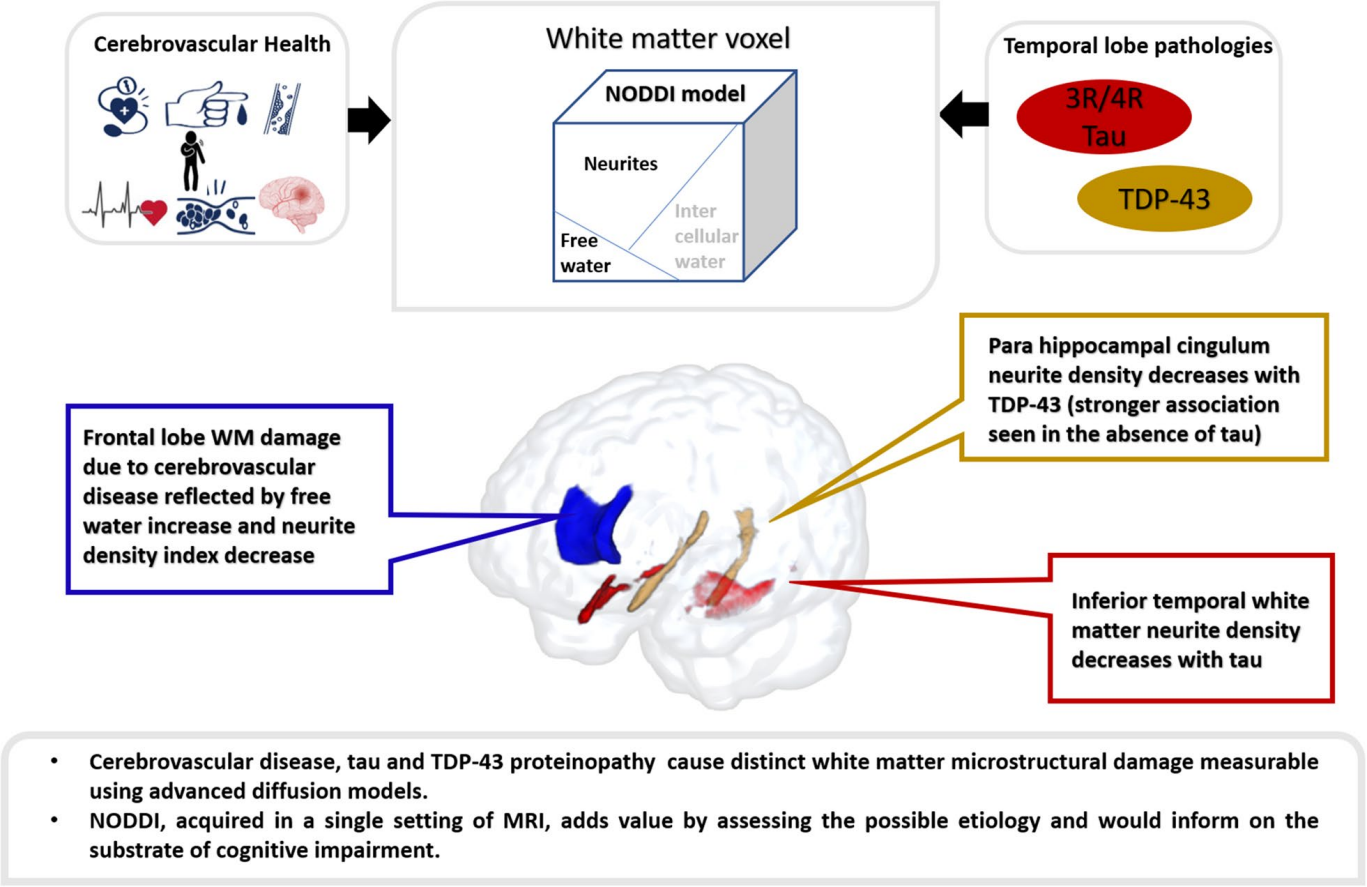

**Supplementary Information:**

The online version contains supplementary material available at 10.1186/s40478-022-01319-6.

## Introduction

The prevalence of neurodegenerative pathologies and cerebrovascular disease (CVD) increases with age [1]. Although Alzheimer’s disease (AD) and CVD are the major contributors of cognitive decline in the elderly and are widely studied, evidence suggests the co-occurrence of medial temporal lobe (MTL) changes are caused by the TAR DNA binding protein of 43 kDa (TDP-43) [2–4], also recently referred to as limbic-predominant age-related TDP-43 encephalopathy (LATE) [5]. Typically, gray matter changes due to these age-related pathologies are the focus of most aging and dementia imaging studies. However, white matter (WM) damage associated with these pathological changes and their relevance to cognitive performance are not widely considered except for visible WM damage such as white matter hyperintensities.

Systemic vascular health and CVD have widespread impact on WM health [6, 7]. Greater damage has been observed in the frontal lobes related to anterior–posterior gradient in aging [8] and in CVD [9]. Our studies have also shown that frontal fibers are more vulnerable to age-related and CVD related changes [6, 10] and that the measurement of diffusion properties in the genu of the corpus callosum (we herein refer to this tract as “Genu”) captures WM damage primarily characterized by aging and CVD. On the other hand, pathological hallmark features of AD accumulation of two proteins, extracellular amyloid-β (Aβ) and intracellular neurofibrillary tangles composed of hyperphosphorylated tau [11] have a differential impact on WM health. Aβ accumulation has minimal effects on WM health [12, 13]. In contrast, temporal WM tracts may not only aid in the spreading of tau [14] but are progressively damaged because of increasing local tau toxicity [15]. Two tracts that are of particular interest in the context of tau are the parahippocampal cingulum bundle (commonly referred to as CGH – cingulum adjoining the hippocampus) [14] and inferior temporal WM (ITWM) [16]. The CGH WM tract is the primary connection with the hippocampus which is one of the earliest regions of tau deposition. The inferior temporal lobe tau which is affected in Braak stages III-IV; has high tau burden along the AD continuum [17].

The increasing accumulation of TDP-43 in the temporal lobes may also be hypothesized to cause damage to both CGH and ITWM tracts. While the gold standard for TDP-43 is an autopsy, recent evidence suggested [18F]-fluorodeoxyglucose PET (FDG-PET) derived inferior/medial temporal (IMT) hypometabolism as a diagnostic marker of TDP-43 proteinopathy associated hippocampal sclerosis aging [18]. A more recent study reported higher sensitivity and specificity of IMT to frontal supra orbital ratio (IMT/FSO) for predicting earlier stages of AD-related TDP-43(+) status [19]. One could hypothesize that the TDP-43 proteinopathy damages white matter in the temporal lobes in addition to neuronal dysfunction observed on FDG-PET.

Traditionally, diffusion tensor imaging (DTI) has been used extensively to study WM changes due to AD and CVD and their association with cognition [20, 21]. However, the DTI findings are generally limited by their inability to accurately capture the nature of underlying tissue properties and their overestimation of voxels within crossing fibers [22, 23]. The recently developed Neurite and Orientation Dispersion Density Imaging (NODDI) model is a biophysically inspired method that provides a much more appropriate model for the estimation of biological measures using diffusion images [24] and enhances DTI findings by separating out free water contribution from the microstructural tissue compartment. NODDI model can estimate the Neurite Density Index (NDI, nominally the proxy for axonal density), Orientation Dispersion Index (ODI, an indicator of the lack of axonal alignment) and Isotropic Volume Fraction (ISOVF, the fraction of water diffusing freely without encountering membranes). To date, there is evidence that NODDI can be used to identify WM changes in aging [25, 26], neurodegenerative diseases [27–29], and associated cognitive deficits [29, 30]. These NODDI measures also can provide information about neurite morphology and have shown sensitivity to tau [16]. Evidence from rodent’s tau model showed reduced NDI in WM and hippocampus [28]. Additionally, reduced cortical NDI in temporal and frontal lobes have been shown [31, 32], and reduced NDI in the cortex was associated with worse cognitive performance in AD [29, 31, 32]. We reported recently that NODDI derived indices (NDI and ISOVF) are sensitive to age and disease related WM damage and cognition [33].

Building on our previous work, in this paper we leveraged NODDI (NDI and ISOVF) measurements for evaluating aging and pathology related WM changes in the brain. Our central hypothesis was that NODDI measures from three WM tracts (Genu, ITWM, and CGH) would aid in differentially capturing WM damage due to CVD, tau, and TDP-43 pathologies and explaining variance in cognition in both the population-based cohort of Mayo Clinic Study of Aging (MCSA) and among clinically diagnosed AD dementia cases enrolled in the Mayo Alzheimer’s Disease Research Center (ADRC). To test our hypothesis, we conducted two sets of analyses. First, we evaluated the association of vascular health, AD and TDP-43 biomarkers, and NODDI measurements in these three tracts. Second, we measured and compared the contribution of these NODDI measures for cognitive decline and compared this to amyloid and tau PET in the MCSA and ADRC cohorts.

## Materials and methods

### Selection of participants

Study participants were selected from the MCSA and ADRC. The selection criteria were the availability of multi-shell diffusion MRI (NODDI), amyloid and tau PET scans, vascular health indicators, and complete clinical assessments. FDG-PET was available in a subset of participants.

#### MCSA participants

The MCSA is an epidemiological cohort designed to investigate the prevalence, incidence, and risk factors for mild cognitive impairment (MCI) and dementia among the residents of Olmsted County, Minnesota [34]. The Olmsted county population was enumerated using Rochester Epidemiology Project (REP) medical records linkage system infrastructure [35, 36] and participants were randomly invited to participate in the MCSA using an age- and sex-stratified sampling frame. The clinical diagnosis of the participants was ascertained at the time of MRI using previously published criteria [37]. Based on the selection criteria, we included 347 participants from the MCSA (> 60 years) with the following clinical characteristics: 281 cognitively unimpaired, 61 MCI, and 5 dementia participants. Because all MCSA participants were recruited from the REP, we were able to include nurse abstracted vascular health indicators from the medical records—hypertension, diabetes mellitus, dyslipidemia, cardiac arrhythmias, coronary artery disease, congestive heart failure, and stroke to compute the composite score (CMC) as published previously [38].

#### ADRC participants

We included 61 amyloid positive clinically diagnosed AD dementia participants confirmed through consensus criteria from the Mayo Clinic-Rochester ADRC who were aged ≥ 65 years to capture typical AD.

#### Standard protocol approvals, registrations, and patient consents

The study was approved by the Mayo Clinic and Olmsted Medical Center institutional review boards and written informed consent was obtained from all participants or their qualified caregivers.

### Imaging

#### MRI acquisition and processing

All MRIs were acquired on 3 T Siemens Prisma scanners using 64-channel receiver head coils. The acquisition protocols and analysis have been published previously for the MCSA [33]. All structural scans were acquired using magnetization prepared rapid gradient echo (MPRAGE) sequence with following parameters: TR = 2300 ms, TE = 3.14 ms, TI = 945 ms, flip angle = 9°, and isotropic resolution = 0.8 mm. The diffusion scans were obtained with multi-band sequence with following parameters: TR = 3400 ms, TE = 71 ms, field of view = 232 mm, and voxel size = 2.0 mm isotropic. The diffusion data consisted of 127 volumes with 13 non-diffusion-weighted images (b = 0 s/mm^2^), and 114 diffusion-encoding gradient directions (6 b = 500, 48 b = 1000, and 60 b = 2000s/mm^2^). The data were evenly spread over the entire spherical shells using an electrostatic repulsion model [39]. Then, the diffusion images were processed as previously described (Raghavan et al. 2021). Briefly, the diffusion-weighted images were denoised [40], corrected for head motion, eddy current distortion [41], and Gibbs ringing [42], then debiased [43]. The diffusion tensors were then fit using a nonlinear least squares fitting algorithm implemented in dipy [44]. NDI, and ISOVF maps were estimated using the Accelerated Microstructure Imaging via Convex Optimization (AMICO) implementation [45] of NODDI in Python. Our recent work showed a more consistent relationship of aging pathologies and cognition with NDI and ISOVF but not ODI [33] therefore we limited the analyses to NDI and ISOVF in this work. Median values of NODDI measures in the Genu, CGH, and ITWM were obtained by non-linearly registering the JHU “Eve” WM atlas [46] to each participant. The registration was calculated by aligning the “Eve” FA image to the participant FA using ANTS [47].

#### Amyloid, tau, and FDG PET markers

The acquisition, processing, and calculations of amyloid, tau, and FDG PET standardized uptake value ratio (SUVR) was described previously [18, 48]. Global amyloid SUVR for each participant was computed by calculating the median uptake over voxels in the prefrontal, orbitofrontal, parietal, temporal, anterior cingulate, and posterior cingulate/precuneus regions normalized by the median amyloid PET uptake in the cerebellar crus grey matter. Global tau SUVR for each participant was computed by calculating median tau PET uptake in the entorhinal, amygdala, parahippocampal, fusiform, inferior temporal, and middle temporal regions normalized by the median tau PET uptake in the cerebellar crus grey matter. We used continuous amyloid PET and tau PET as our primary AD biomarkers. We also utilized amyloid positivity (amyloid cutoff for positivity was SUVR ≥ 1.48) and tau positivity (tau cutoff for positivity was SUVR ≥ 1.25) established previously [48] for additional analyses.

For FDG-PET, we selected 3 regions of interest (ROI) from the ADIR122 atlas (available as part of the Mayo Clinic Adult Lifespan Template, nitrc.org/projects/mcalt/) which have been associated with TDP-43 positivity: inferior temporal (IT), medial temporal (MT) (amygdala and hippocampus), and frontal supraorbital (FSO). Regional FDG SUVR for each participant was computed by calculating the median uptake from gray matter voxels in each ROI normalized by the median FDG uptake in the pons. Then, we assessed the ratio of IT to MT (IMT) and then IMT to FSO (IMT/FSO) and used as our FDG TDP-43 signature as this was proposed previously as a biomarker of TDP-43(+) status [19].

#### Neuropathologic analysis

Autopsy analysis was conducted in 9 deceased participants using a standardized dissection and sampling procedure recommended by Consortium to Establish a Registry for Alzheimer's Disease (CERAD) [49]. Formalin-fixed and paraffin-embedded left hemisphere brain sections were evaluated and were used for immunohistochemical analysis of TDP-43 (pS409/410, 1:10,000, Cosmo Bio). Cases were considered TDP-43(+) if TDP-43 immunoreactive neuronal cytoplasmic inclusions, perivascular bi-lobed inclusions, or neuronal intranuclear inclusions were observed in the amygdala [50, 51].

### Cognitive performance

The MCSA neuropsychological battery consists of nine tests covering 4 cognitive domains, as previously published [34, 37]. The primary outcome for the analysis was a global cognitive z score that was estimated from the z-transformation of the average of the 4 cognitive domain z scores (memory, language, attention/executive, and visuo-spatial function) [52]. Across MCSA and ADRC studies, the common test was Mini-Mental State Examination (MMSE) [53], estimated from Short test of Mental Status [54], which we evaluated as an outcome for comparison.

### Statistical analyses

The participant characteristics were summarized using mean (standard deviation, SD) for the continuous variables and count (%) for the categorical variables. Amyloid and tau distribution were skewed and hence analyzed with a log transformation.

Pearson partial correlations were used to identify the associations between vascular risk (CMC), AD biomarkers, FDG-TDP-43 signature with our tracts of interests after adjusting for age and sex.

Then, we fit separate multiple linear regression models with cognitive scores as an outcome variable (global cognition and MMSE in MCSA and MMSE in ADRC), with amyloid, tau, and regional diffusion measures as predictors, and adjusting for age, sex, education, and number of clinical visits (number of times a participant had previously completed cognitive testing in the study to account for practice effects). In addition, we fit multiple linear regression models using a combination of least absolute shrinkage and selection operator (LASSO) with alpha = 1 and step-wise approaches to assess the added benefit of WM vascular component (Genu NDI and Genu ISOVF) and temporal WM diffusion metrics (CGH NDI and ITWM NDI) to AD biomarkers (amyloid and tau) for predicting cognitive performance. These models were also adjusted for age, sex, education, and number of clinical visits. We did not perform multiple comparison testing (which would rely on universal null hypothesis) because we did not want to inflate the probability of Type II error [55, 56].

## Results

The characteristics of MCSA and ADRC data sets are shown in Table 1. There were more males and *APOE* ε4 carriers in the ADRC than the MCSA. All participants in the ADRC were amyloid positive with 92% of them were tau positive. Whereas, in the MCSA, 47% were amyloid positive with 36% of them were tau positive.

**Table 1:** Characteristics table of subjects with the mean (SD) listed for the continuous variables and count (%) for the categorical variables

Characteristics	MCSA n = 347	ADRC n = 61
*Demographics and cognition*		
Age, yrs	74.3 (8.5)	74.4 (6.0)
Males, no. (%)	185 (53%)	36 (59%)
APOE4, no. (%)	101 (31%)	32 (71%)
Education, yrs	15.0 (2.6)	16.0 (2.7)
MMSE	28.3 (1.8)	21.9 (5.0)
zGlobal	− 0.02 (1.41)	NA
CDR	0.2 (0.7)	4.1 (2.9)
Cognitively Impaired, no. (%)	66 (19%)	61 (100%)
*CVD markers*		
WMH	1.03 (1.04)	1.35 (1.19)
Hypertension, no. (%)	221 (64%)	27 (44%)
Diabetes, no. (%)	56 (16%)	5 (8%)
Dyslipidemia, no. (%)	281 (81%)	21 (34%)
*AD and TDP-43 markers*		
PIB SUVr	1.67 (0.48)	2.43 (0.44)
Tau SUVr	1.23 (0.14)	1.78 (0.46)
FDG TDP-43^a^	0.83 (0.06)	0.87 (0.12)
*Diffusion Markers (NODDI and DTI models)*		
Genu ISOVF	0.11 (0.03)	0.13 (0.03)
Genu NDI	0.54 (0.04)	0.52 (0.04)
ITWM NDI	0.49 (0.04)	0.47 (0.03)
CGH NDI	0.49 (0.03)	0.46 (0.03)

CDR, clinical dementia rating scale; WMH, white matter hyperintensity; SUVR, standard uptake value ratio; TDP-43, trans-active response DNA-binding protein of 43; ISOVF, isotropic volume fraction; CGH, parahippocampal cingulum; ITWM, inferior temporal white matter, NDI, neurite density index

^a^Eighty-eight participants from MCSA and fourteen participants from ADRC were missing FDG PET scans

### NODDI measurements were associated with vascular risk factors, amyloid, tau, and TDP-43

**Fig. 1:**
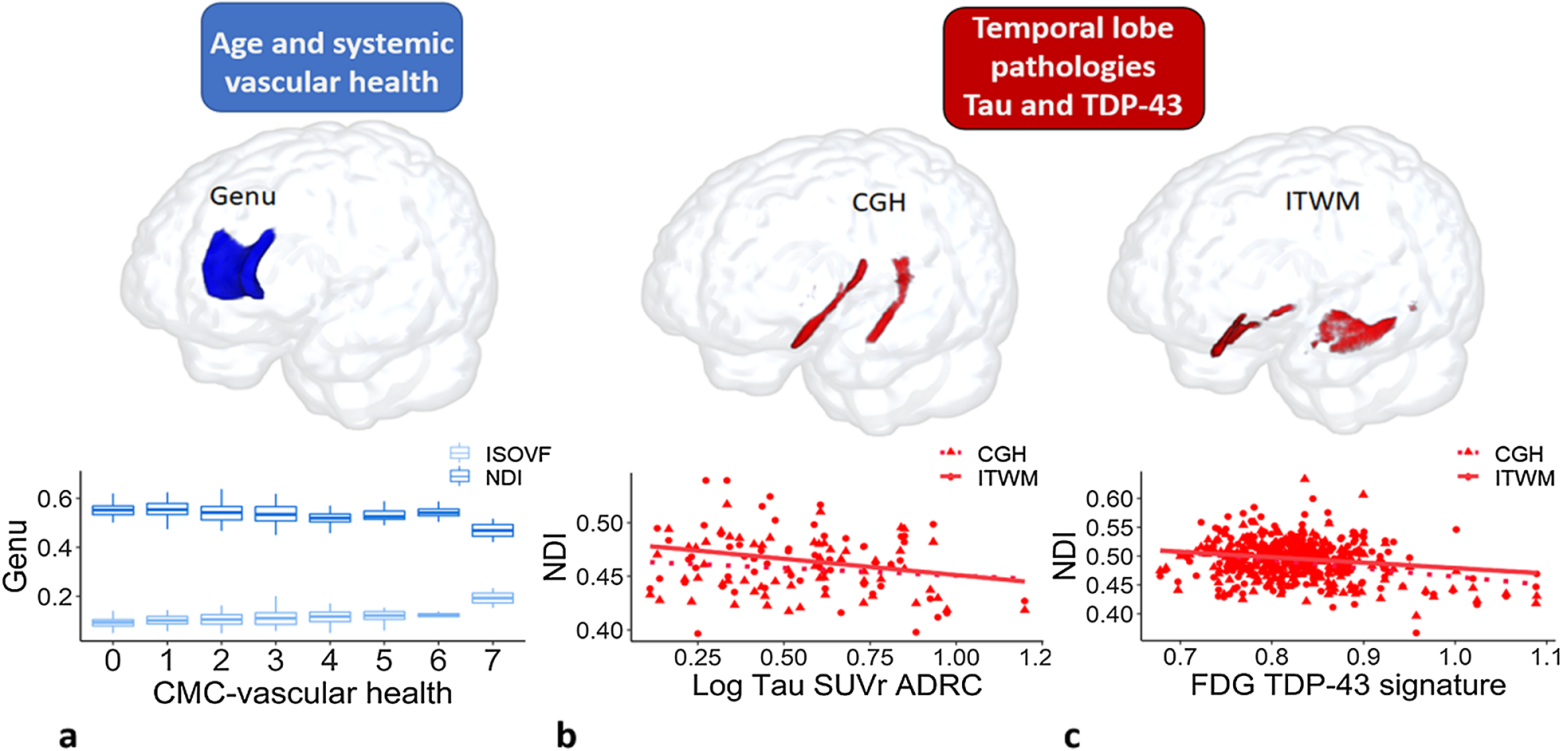
Conceptual diagram illustrating the white matter tracts studied and their biological associations. Top panel: Genu as the white matter tract studied for systemic vascular health; parahippocampal cingulum (CGH) and inferior temporal white matter (ITWM) studied for temporal lobe pathologies. **A** Association of Genu white matter NODDI measures with systemic vascular health in MCSA. **B** Association of CGH and ITWM NDI measures with tau deposition in ADRC. Tau values were log transformed. **C** Association of CGH and ITWM NDI measures with FDG TDP-43 signature in MCSA. Inferior temporal to medial temporal (IMT) and frontal supra orbital ratio (IMT/FSO) was used as FDG TDP-43 signature

As explained in the introduction, the present study confirmed the relevance of Genu WM changes to cerebrovascular health. Both Genu ISOVF (r = 0.15, *P* = 0.005) and Genu NDI (r = − 0.15, *P* = 0.005) were correlated with vascular risk measure i.e. CMC. A plot of the Genu NODDI measures with CMC in MCSA are shown in the first panel of Fig. 1. Therefore, we used both Genu ISOVF and Genu NDI as the components of CVD (WM vascular component). Amyloidosis was associated with an increase in Genu ISOVF (r = 0.13, *P* = 0.02) in the MCSA, but none of the partial correlations were significant in the ADRC.

As stated in the introduction, both ITWM and CGH are likely damaged by tau and TDP-43 such that loss of synapses and axons would be reflected in worsening neurite density on NODDI. Therefore, we considered these two tracts as our temporal WM component. In ADRC, where there is a greater range of tau, we found evidence that neurite density (correlate of loss of synapses and neurons) in the ITWM was marginally associated with tau (r = − 0.24, *P* = 0.07 for ITWM). This is illustrated in the middle panel of Fig. 1. Using the FDG-PET TDP-43 signature, we found that reduced NDI in the CGH of MCSA (n = 259) was associated with increased FDG-PET TDP-43 signature (r = − 0.23, *P* < 0.001 for CGH NDI). This is shown in the third panel of Fig. 1. We also performed a sensitivity analysis in a subset of amyloid negative 80 + MCSA participants (n = 22 of which 17 were cognitively unimpaired and 5 were impaired), who are also likely to have low WM damage in association with tau and are at a high risk of TDP-43. We found a significant association of NDI in CGH with TDP-43 signature (r = − 0.59, *P* = 0.007). We did not find any associations of ISOVF in temporal tracts with disease biomarkers, so we did not consider ISOVF in the temporal WM component for further analyses.

### Relevance of NODDI in the context of AD biomarkers

**Fig. 2:**
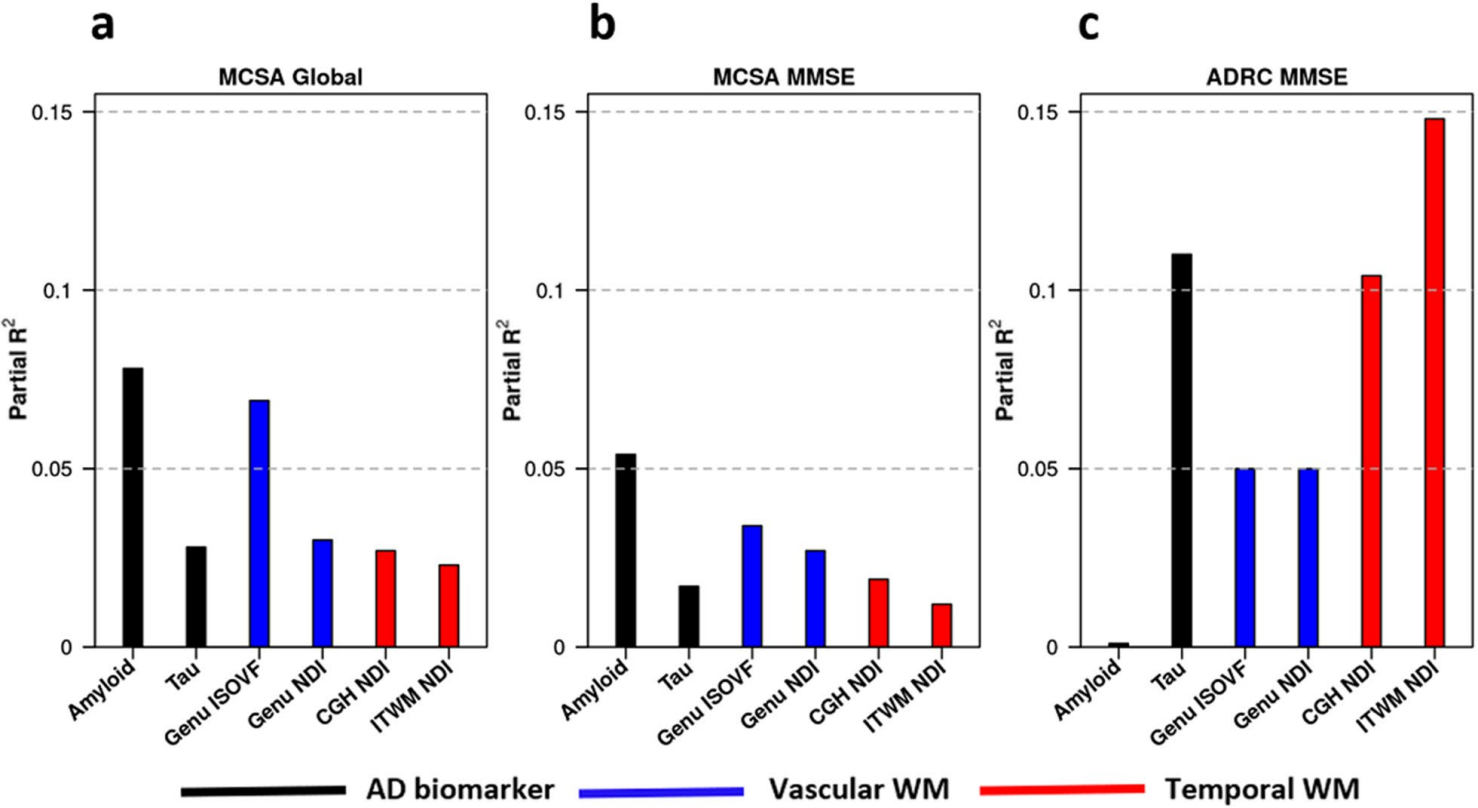
Relative univariate contribution of imaging biomarkers to cognitive performance. ISOVF, isotropic volume fraction; NDI, neurite density index; CGH, parahippocampal cingulum; ITWM, inferior temporal white matter; MMSE, mini mental state examination

The models for both MCSA and ADRC samples showing an association between the individual neuroimaging measures and cognitive performance after adjusting for age, sex, education, and number of clinical visits (single biomarker models) are shown in Additional file 1: Table S1 and also in Fig. 2. To identify the contribution of WM NODDI measures to overall cognitive performance, we considered global cognition as a primary outcome in the MCSA. In MCSA, amyloid, tau, and all diffusion metrics showed a significant contribution to global cognition and MMSE when each factor was considered independently. Among the diffusion metrics, higher Genu ISOVF was most strongly associated with lower cognitive performance. We also observed a greater predictive power for Genu ISOVF (partial R^2^ = 6.9% for global cognition and 3.4% for MMSE) than Genu NDI (partial R^2^ = 3% for global cognition and 2.7% for MMSE) in Fig. 2. Tau was a relatively lesser predictor of cognitive performance in this community dwelling cohort owing to its broad age ranges and relatively lower frequency of tau positivity.

In the ADRC cohort of amyloid positive individuals, higher ITWM NDI had a better prediction for lower cognitive performance than tau (14.8% and 11% respectively) univariately.

### NODDI and AD biomarkers for detection of possible etiology and prediction of cognitive performance

The final models that included all imaging biomarkers (except FDG-PET TDP-43 signature), age, sex, education, and number of clinical visits for predicting cognitive performance are shown in Table 2. Older age and male sex were associated with worse global cognitive performance in the MCSA. In both MCSA the global cognition and MMSE models, higher education was associated with better cognition. There was contribution of amyloid, Genu ISOVF, and Genu NDI to worse global cognition and MMSE. However, amyloid contribution to global cognition (7.4%) was comparable to MMSE (6.6%). After accounting for amyloidosis, there was no significant contribution of the WM temporal component with both global cognition and MMSE. In the ADRC sample, only two predictors emerged as important. Lower tau (partial R^2^ = 0.10) and higher NDI in ITWM predicted better MMSE performance **(**partial R^2^ = 0.09).

**Table 2:** Final parsimonious models evaluating the utility of neuroimaging measures in predicting cognitive performance

Variable	Estimate (s.e.)	*p* value	Partial R^2^
*MCSA Global Cognition (Model R*^2^ = 0.546)			
Intercept	0.48 (1.08)	0.66	
Age	− 0.06 (0.009)	< 0.001	0.121
Male	− 0.23 (0.10)	0.031	0.014
Education	0.17 (0.02)	< 0.001	0.178
Visit Number	0.11 (0.02)	< 0.001	0.090
Amyloid	− 1.23 (0.24)	< 0.001	0.074
Genu ISOVF	− 9.16 (2.20)	< 0.001	0.049
Genu NDI	4.65 (1.35)	< 0.001	0.034
*MCSA MMSE (Model R*^*2*^* = 0.282)*			
Intercept	23.26 (1.35)	< 0.001	
Education	0.19 (0.03)	< 0.001	0.092
Amyloid	− 1.78 (0.36)	< 0.001	0.066
Genu ISOVF	− 11.91 (3.12)	< 0.001	0.041
Genu NDI	8.01 (2.09)	< 0.001	0.041
*ADRC MMSE (Model R*^*2*^* = 0.209)*			
Intercept	4.15 (9.45)	0.66	
Tau	− 5.99 (2.41)	0.016	0.096
ITWM NDI	45.08 (19.40)	0.024	0.085

ISOVF, isotropic volume fraction; CGH, parahippocampal cingulum; ITWM, inferior temporal white matter, NDI, neurite density index, MMSE, mini mental state examination. The initial had all NODDI measures but only these variables

Significant predictors of cognition are shown in the parsimonious models. The models included all of these as potential predictors: age, male, education, visit number, amyloid, tau, genu ISOVF, genu NDI, ITWM NDI, and ITWM CGH

**Fig. 3:**
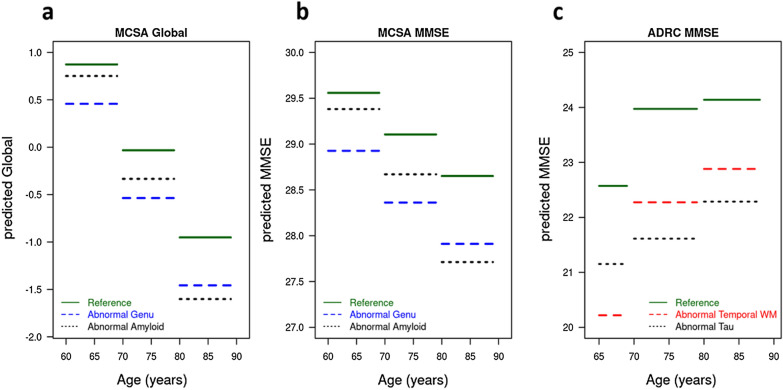
The predicted cognition by age group for a given value of NODDI measure and AD biomarker. The reference (green) lines are predictions for healthy white matter tracts and low AD biomarkers (low amyloid, low tau, high temporal NDI, high Genu NDI, low Genu ISOVF). The blue lines are predictions for poor Genu health (high Genu ISOVF and low Genu NDI) in MCSA. The black lines are predictions for abnormal AD biomarker (high amyloid in MCSA and high tau in ADRC). The abnormal temporal WM (red) lines in ADRC show predictions for low ITWM. In the plot, reference (low) and abnormal (high) are defined by 25th and 75th percentiles

Figure 3 shows the predicted cognition by age group in MCSA and ADRC samples. Here, normal and abnormal were defined in quartiles with 25th and 75th percentiles of the measures within each age group respectively. For MCSA global cognition, the rapid lowering of predicted cognition as a function of age was expected given that age explains 12% of variance in cognition (Table 2) and the frequency of pathologies increases with age. For MCSA MMSE performance (middle panel), the average predicted MMSE was above 29 until age 70 after which there was a visible decline in the baseline MMSE which is typically observed in the population due to increasing burden of age-related pathologies. For the ADRC MMSE results in Fig. 3, there was worse MMSE in younger AD patients because more aggressive disease trajectories have been observed in younger AD patients [57].

### Neuropathology findings as indicators of TDP-43 status:

Interestingly, a small subset (n = 9) had autopsy findings. Though the samples are small, NODDI data compared against gold-standard pathology provides insights into variability of NODDI as a function of pathological burden specifically TDP-43. Three out of four MCSA cases (75%) and two out of 5 (40%) ADRC cases were TDP-43(+). The imaging and neuropathological characteristics of the autopsy cohort are shown in Table 3 and discussed further below. The first TDP-43 positive case in MCSA (case 1) was hypertensive with a greater number of cardiovascular and metabolic conditions, higher Genu ISOVF, elevated IMT/FSO hypometabolism ratio, lower ITWM and CGH NDI reflecting the contribution of CVD and temporal WM contribution to impairment. Similarly, case 2 was tau-PET negative with hypertension, higher Genu ISOVF, high IMT/FSO hypometabolism ratio, very low ITWM and CGH NDI highlighting the usefulness of NODDI in helping assess vascular health. ADRC participants generally had low vascular disease burden but a greater extent of MTL damage (low Genu ISOVF, low ITWM, and low CGH NDI) which was reflected in the ADRC final model. Except one control, all other TDP-43(+) cases had abnormal hippocampal volume.

**Table 3:** Characteristics of autopsy cases in our dataset with TDP-43 information available

	Cohort	Age/sex	Clinical diagnosis	Hypertension (CMC^a^ in MCSA)	Amyloid PET (+)^b^ (SUVR)	Tau PET (+)^c^ (SUVR)	Neurodege-neration (+)^d^ (HVa)	TDP-43 (+)^e^ (FDG TDP-43 signature IMT/FSO)	Genu (ISOVF, NDI)	ITWM NDI	CGH NDI	MMSE
**Case 1**	MCSA	85M	Multi-domain MCI	+ (4)	− (1.28)	− (1.24)	+ (− 1.48)	+ (0.81)	0.22, 0.49	0.45	0.48	27
**Case 2**	MCSA	79M	Amnestic MCI	+ (2)	+ (2.12)	− (1.19)	+ (− 1.72)	+ (0.96)	0.19, 0.42	0.37	0.39	26
Case 3	MCSA	83M	Control	+ (2)	− (1.3)	− (1.16)	− (− 0.10)	+ (1.09)	0.23, 0.48	0.45	0.42	30
Case 4	MCSA	82F	Dementia	+ (2)	+ (2.92)	+ (1.73)	+ (− 1.44)	−	0.22, 0.52	0.44	0.46	24
Case 5	ADRC	81M	AD	+	+ (2.41)	+ (1.73)	+ (− 1.88)	− (0.90)	0.22, 0.46	0.45	0.42	23
Case 6	ADRC	74F	AD	−	+ (2.16)	− (1.15)	+ (− 0.91)	− (0.76)	0.11, 0.46	0.45	0.43	14
Case 7	ADRC	80M	AD	−	+ (2.98)	+ (2.08)	+ (− 3.21)	+	0.17, 0.59	0.48	0.45	20
Case 8	ADRC	75M	AD	−	+ (2.68)	+ (1.47)	+ (− 1.83)	− (0.96)	0.18, 0.51	0.47	0.45	22
Case 9	ADRC	76M	AD	+	+ (2.44)	+ (2.31)	+ (− 2.04)	+ (0.96)	0.26, 0.53	0.47	0.46	20

^a^CMC—cardiovascular and metabolic conditions by ascertainment of seven conditions from REP: hypertension, hyperlipidemia, cardiac arrhythmias, coronary artery disease, congestive heart failure, diabetes mellitus, and stroke

^b^Amyloid positivity (+) = SUVR ≥ 1.48; SUVR—standard uptake value ratio

^c^Tau positivity (+) = SUVR ≥ 1.25

^d^Neurodegeneration positivity (+) = Hippocampal volume adjusted for TIV (HVa) from MRI ≤ − 0.76

^e^TDP-43 positivity (+) = presence of TDP-43 immunoreactive neuronal cytoplasmic inclusions, dystrophic neurites, or neuronal intranuclear inclusions in the amygdala in autopsy brain; TDP-43 = trans-active response DNA-binding protein of 43 kDa

ISOVF, isotropic volume fraction; CGH, parahippocampal cingulum; ITWM, inferior temporal white matter, NDI, neurite density index, IMT/FSO, inferior temporal to medial temporal (IMT) to frontal supra orbital (FSO) ratio

Mean (SD) NODDI measures among the populations investigated: For MCSA: Genu ISOVF = 0.11(0.03), Genu NDI = 0.54(0.04), CGH NDI = 0.49 (0.03), ITWM NDI = 0.49(0.04) and for ADRC: Genu ISOVF = 0.13(0.03), Genu NDI = 0.52(0.04), CGH NDI = 0.46 (0.03), ITWM NDI = 0.47(0.03))

## Discussion

In this study, we assessed WM damage using NODDI measures in the frontal and temporal lobes and evaluated their contribution to cognitive decline in two independent data sets. The main conclusions were: (i) Both Genu ISOVF and Genu NDI were sensitive to capturing age and systemic vascular health related WM damage and contributed to the prediction of cognitive performance. The utility of Genu ISOVF was greater in the population-based cohort in comparison to the clinically diagnosed AD dementia cohort with elevated Aβ where there was lower vascular contribution to cognitive impairment. (ii) Neurite density measures in the temporal lobe tracts captured WM changes due to temporal lobe focused pathologies which was associated with tau PET and FDG-PET based TDP-43 signature. (iv) NODDI imaging acquired in the single setting of MRI would add value in assessing the possible etiology (CVD vs. tau related changes vs. possibly TDP-43 in the absence of amyloid and tau) and their corresponding contribution to cognitive performance.

### WM damage and CVD pathologies and its relevance for predicting cognition

We and others have shown that vascular risk factors are associated with poor WM health [6, 21, 58], particularly reduced Genu FA with hypertension [58–60], diabetes [61, 62], and dyslipidemia [63]. However, the FA metric cannot accurately disentangle the contribution from tissue degeneration (axonal damage and myelin breakdown) and free water increase, and NODDI helped us separate these factors using NDI and ISOVF. Our recent study showed that white matter hyperintensities were the strongest predictors of diffusion changes (ISOVF) in addition to age [33]. In this study, we further report that Genu ISOVF and Genu NDI was associated with systemic vascular health in the population-based cohort. Additionally, previous studies observed increased free water changes in the normal-appearing WM using free water elimination method [64–66], suggesting that mechanisms of microvascular degeneration and neuroinflammation due to blood–brain barrier dysfunction contribute to mild vascular damage in small vessel disease and AD.

In the present study, we found a significant contribution of Genu ISOVF and Genu NDI on global cognitive performance and MMSE in the general population along with amyloid (Figs. 2, 3). Previous studies including ours [6, 10] have shown the association of reduced WM integrity in the Genu and worse cognitive performance [67–69]. Our previous study showed that NODDI derived free water or ISOVF in the corpus callosum was a significant predictor of cognitive performance highlighting its usefulness in capturing vascular contribution to cognitive impairment [33]. Notably, as shown in Figs. 2 and 3, Genu was not a significant predictor of MMSE in the AD cohort where the vascular pathology was comparatively less. Our findings support the utility of NODDI for capturing vascular contributions to cognition in populations where there is a greater prevalence of vascular disease contributing to cognitive impairment.

The relevance of NODDI measures for the diagnosis of vascular etiology can also be deduced from Table 3. Case 1 was an 85-year-old male with a clinical presentation of multi-domain MCI (MMSE = 27) with amyloid and tau PET negative. Both the higher number of cardiovascular and metabolic conditions (4 out of 7 possible) and very high (or worse) Genu ISOVF of 0.22 (mean and standard deviation in MCSA were 0.11 and 0.03) and low Genu NDI of 0.49 (mean and standard deviation in MCSA were 0.54 and 0.04) indicates that the vascular contribution to impairment was significant and not sufficiently considered for diagnosis. Similarly, case 2, an 79-year-old male with amnestic MCI, was amyloid positive and tau PET negative. The high Genu ISOVF and low Genu NDI clearly supports the vascular contribution to cognitive impairment. Contrasting cases 5–8 from the ADRC, the lower values of Genu ISOVF and higher Genu NDI explain that the lower extent of vascular disease in the AD cases. While the self-report of CMC was available, the continuous scale of Genu ISOVF reflects the true extent of biological damage.

### Temporal white matter damage and tau and its relevance for predicting cognition

Tau pathology is the underlying cause of axonal and neuronal neurodegeneration in some cognitively normal and in AD dementia individuals [12, 14, 70]. Tau related WM changes often depicts distinct spatial profiles with initial accumulation in temporal lobe regions susceptible to AD pathology and then propagates through axonal projections. Evidence from tau-mouse models showed reduced NDI in WM and hippocampus [28]. In the present study, we showed that in a typical AD population with a greater extent of tau related changes, the temporal NDI (ITWM) measures were useful in detecting tau related diffusion changes. Here, we extend previous observations that reported an association between DTI and NFT pathology in the MTL limbic connections and medial parietal WM [12]. Evidence also suggests the association between tau PET and anterior temporal cortex and associated pathways [70], suggesting greater WM vulnerability in tracts associated with early tau accumulation [29, 71].

CGH is an important tract connecting the hippocampus to the posterior cingulate cortex, and it has been reported that CGH diffusivity significantly predicts downstream tau accumulation and associated memory decline in amyloid positive individuals [14]. More recently, Wen et al. [16] studied the spatial pattern of tau propagation using a DTI and NODDI derived data-driven method and suggested reduced axonal packing density and higher diffusion freedom in white matter in regions of tau deposition (which included inferior temporal to inferior parietal regions in amyloid positive participants). Additionally, studies have shown reduced cortical NDI in temporal and frontal lobes [31, 32] which is suggested to be associated with worse cognitive performance in AD [29, 31, 32]. In our data, white matter temporal component NDI predicted MMSE and global cognitive performance, suggesting that these regional metrics could be sensitive to tau mediated WM damage and hence would be a relevant biomarker for studying disease progression. When we focused on multivariable analyses using a combination of Lasso and stepwise regression, tau and ITWM NDI emerged as the significant predictors of cognitive performance for typical AD. This was supported by recent in vivo PET studies that demonstrated a more extensive reduction in synaptic density in the medial temporal and neocortical early AD brains [72, 73]. Furthermore, reduced cortical NDI and ODI in the mesial and lateral temporal lobes of AD brains were associated with increased ^18^F-THK5351 signal and worse cognitive performance, implicating the role of tau and neuroinflammatory pathology in the formation of abnormal neuritis [74]. Notably, our observed non-trivial contribution of the NDI in ITWM (early neocortical pathway associated to tau deposition in AD) to cognitive performance along with tau suggests the integral role of NODDI as an early marker of cognitive changes in the preclinical stages (clearly shown in the third panel of Fig. 3)

Amyloid was useful in predicting cognition in the community cohort because there is a greater range of amyloidosis across the population and lower frequency of high levels of tau burden. However, the multivariable analysis in the MCSA not retained the effect of NDI in temporal tracts suggesting that temporal WM health is more strongly associated with tau than vascular etiology (Fig. 2). On the other hand, tau PET information and tau-mediated white matter injury (reflected with worsening ITWM NDI) predicted memory performance in the ADRC. Figure 3 illustrates how specific NODDI measures can be used to predict cognition along with AD biomarkers in two independent populations and the extent of damage on NODDI can also reflect the extent of damage attributed to CVD vs. tau-specific damage.

### Temporal white matter damage and TDP-43 and its relevance for predicting cognition

The co-existence of multiple pathologies along with AD neuropathological changes especially in individuals after 80 years of age adds to the prediction of worsening cognitive performance. Clinical and neuropathological evidence identified accumulation of TDP-43 inclusions in association with AD [2, 3] and hippocampal sclerosis [75], suggesting it is a major contributor to cognitive dysfunction. Also, TDP-43 exacerbates the memory impairment and cognitive decline in individuals with AD pathologies [3, 76]. While there is no definite biomarker for TDP-43 other than autopsy, predominant involvement of MTL structures [4, 5] and recent evidence supports the usefulness of a FDG-PET hypometabolic signature as being correlated with the presence of TDP-43 [18, 19]. This proposed FDG TDP-43 marker, the derived IMT or IMT/FSO ratio, reliably identified the tau-negative amnestic status in older individuals [18, 19]. The association we observed between CGH NDI and FDG IMT/FSO ratio in all MCSA and amyloid negative 80 plus individuals suggests that neurite density also changes with other prominent non-AD temporal lobe pathology such as TDP-43.

Pathology information sheds additional light on the relevance of NODDI temporal WM values for identifying the likely etiology in Table 3. Cases 1–3 were classified as TDP-43(+) at autopsy. In all three cases, the tau PET scans were also negative, but the temporal WM measure was abnormal because the values were lower than 0.49 (average for CGH and ITWM in MCSA). The worse NDI in these tracts that are spread across the temporal lobes illustrates the likelihood that a different etiology such as TDP-43 or PART [77] (primary age-related tauopathy refers tau in the absence of amyloidosis) may have contributed to the cognitive impairment. As reported previously [18, 78], the current TDP-43(+) participants had abnormal hippocampal volumes, suggesting that both TDP-43 and hippocampal sclerosis accounted the tau-negative amnestic syndrome. While the NODDI measures also have the same non-specificity of a medial temporal atrophy [79] measure, future studies in larger datasets with autopsy confirmed data will be able to shed light on the usefulness of NODDI for identifying the underlying etiology of cognitive impairment. It cannot be ruled out that the WM changes observed may be due to PART, which will need confirmation in a larger autopsy cohort.

### Usefulness of NODDI in Aging and Dementia Studies

Overall, our findings support the utility of non-invasive, increasingly available, and affordable biophysical diffusion models as proxy measures for WM damage due to vascular, AD, and TDP-43. As multi-band acceleration has become commonplace, these measures can inform the likely substrate of cognitive impairment and can facilitate early detection and tracking of disease progression in aging and dementia studies. Future studies will build on this work to optimize the usage of NODDI for differential diagnosis and expand on the idea of tracking longitudinal WM damage with disease progression.

An important finding from this study is that the usefulness of regional NODDI measures will vary based on the cohort under investigation. For example, in the community-dwelling cohort where multiple pathologies increase with age and contribute to cognitive decline, frontal WM would contribute to the prediction of cognitive performance. On the other hand, in clinically diagnosed AD dementia patients recruited from a dementia clinic where the primary substrate of dementia is likely tau deposition, temporal WM damage related to tau would be a significant predictor of cognitive performance.

## Strength and limitations

A key strength of this study was the rich multimodal imaging information from two independent samples with varying degrees of CVD and AD pathologies along with pathology information in a subset. This allowed us to assess the vascular and AD contributions to diffusion alterations and associated cognitive performance. The use of an advanced diffusion model with conventional DTI enabled us to disentangle the source of WM alterations more accurately with precise control of CSF partial volume effects. Another strength is the focus on multiple cognitive measures in the MCSA sample to evaluate CVD-related cognitive impairment. A major limitation is the relatively modest sample size of typical AD participants in ADRC with comparatively less vascular disease that may limit the tract specific association findings. Another limitation is that we utilized FDG-PET TDP-43 signature as a surrogate for TDP-43 due to the lack of sufficient pathology data and TDP-43 also may likely be present in AD patients. However the association between NODDI and the FDG-PET TDP-43 signature in amyloid negative 80 + participants lends support for our hypothesis that NDI in temporal WM tracts are impacted by TDP-43. Future work based on a larger cohort with multiple biophysical diffusion models, pathology information, and longitudinal imaging measurements will further allow us to expand on these important findings.

## Conclusions

In the present study, we found that cerebrovascular disease, tau, and TDP-43 proteinopathy cause white matter microstructural damage measurable using advanced diffusion NODDI models. In two independent cohorts with different clinical composition, we showed the clinical utility of NODDI models in predicting cognitive performance and its usefulness in identifying the substrate of cognitive impairment. Therefore, NODDI, acquired in a single setting of MRI, can add significant clinical value for differential diagnosis in cognitive impairment and dementia.

## Supplementary Information

**Additional file 1**: **Table S1**. Single biomarker models evaluating the utility of neuroimaging measures for predicting cognitive performance after accounting for age, sex, education, and number of clinical visits.

## Data Availability

The data used in this study will be made available upon reasonable request following MCSA and ADRC study procedures.

## Abbreviations

AD: Alzheimer’s disease
ADRC: Alzheimer’s Disease Research Center
CGH: Parahippocampal cingulum
CSF: Cerebrospinal fluid
CU: Cognitively unimpaired
CVD: Cerebrovascular disease
DTI: Diffusion tensor imaging
FA: Fractional anisotropy
FDG-PET: Fluorodeoxyglucose PET (FDG-PET)
FLAIR: Fluid attenuated inversion recovery
GCC: Genu of corpus callosum
GM: Gray matter
ISOVF: Isotropic volume fraction
ITWM: Inferior temporal white matter
MCI: Mild cognitive impairment
MCSA: Mayo clinic study of aging
MD: Mean diffusivity
MMSE: Mini mental state examination
NDI: Neurite density index
NODDI: Neurite orientation dispersion and density Imaging
ODI: Orientation dispersion index
PET: Positron emission tomography
TDP-43: TAR DNA binding protein of 43 kDa
WM: White matter
