# Supplementary material for: White matter damage due to vascular, tau, and TDP-43 pathologies and its relevance to cognition
Source: Acta Neuropathol Commun. 2022 Feb 5;10:16. doi: 10.1186/s40478-022-01319-6 (PMC8817561; doi:10.1186/s40478-022-01319-6)
Supplement: Supplementary file 1 — Additional file 1: Table S1. Single biomarker models evaluating the utility of neuroimaging measures for predicting cognitive performance after accounting for age, sex, education, and number of clinical visits. [file 40478_2022_1319_MOESM1_ESM.docx]

**Table S1**

Single biomarker models evaluating the utility of neuroimaging measures for predicting cognitive performance after accounting for age, sex, education, and number of clinical visits

|  | | | | |
| --- | --- | --- | --- | --- |
| **Variable** | **Estimate (s.e.)** | **p-value** | Model  **R^2^** | Partial  **R^2^** |
| **MCSA Global cognition** | | | | |
| Amyloid | -1.31 (0.24) | <0.001 | 0.503 | 0.078 |
| Tau | -1.74 (0.56) | 0.002 | 0.476 | 0.028 |
| Genu ISOVF | -11.40 (2.27) | <0.001 | 0.498 | 0.069 |
| CGH NDI | 6.30 (2.04) | 0.002 | 0.475 | 0.027 |
| ITWM NDI | 4.88 (1.74) | 0.005 | 0.473 | 0.023 |
| Genu NDI | 4.67 (1.44) | 0.001 | 0.477 | 0.030 |
| **MCSA MMSE** | | | | |
| Amyloid | -1.70 (0.39) | <0.001 | 0.253 | 0.054 |
| Tau | -2.11 (0.87) | 0.017 | 0.223 | 0.017 |
| Genu ISOVF | -12.44 (3.62) | <0.001 | 0.237 | 0.034 |
| CGH NDI | 8.17 (3.20) | 0.011 | 0.225 | 0.019 |
| ITWM NDI | 5.49 (2.73) | 0.045 | 0.220 | 0.012 |
| Genu NDI | 6.88 (2.25) | 0.002 | 0.231 | 0.027 |
| **ADRC MMSE** | | | | |
| Amyloid | 0.60 (3.40) | 0.86 | 0.124 | 0.001 |
| Tau | -6.80 (2.60) | 0.012 | 0.220 | 0.110 |
| Genu ISOVF | -34.48 (20.17) | 0.093 | 0.168 | 0.050 |
| CGH NDI | 62.01 (24.49) | 0.014 | 0.215 | 0.104 |
| ITWM NDI | 58.62 (18.93) | 0.003 | 0.254 | 0.148 |
| Genu NDI | 30.383 (17.83) | 0.094 | 0.168 | 0.050 |
| ISOVF – isotropic volume fraction; CGH – parahippocampal cingulum; ITWM- inferior temporal white matter, NDI – neurite density index, , MMSE – mini mental state examination. | | | | |
